# Directly Exploring the Neural Correlates of Feedback-Related Reward Saliency and Valence During Real-Time fMRI-Based Neurofeedback

**DOI:** 10.3389/fnhum.2020.578119

**Published:** 2021-02-05

**Authors:** Bruno Direito, Manuel Ramos, João Pereira, Alexandre Sayal, Teresa Sousa, Miguel Castelo-Branco

**Affiliations:** ^1^Coimbra Institute for Biomedical Imaging and Translational Research (CIBIT), University of Coimbra, Coimbra, Portugal; ^2^Institute of Nuclear Sciences Applied to Health (ICNAS), University of Coimbra, Coimbra, Portugal; ^3^Siemens Healthineers, Lisbon, Portugal; ^4^Faculty of Medicine, University of Coimbra, Coimbra, Portugal

**Keywords:** neurofeedback, reward, real-time fMRI (rtfMRI), volitional modulation, adaptive threshold

## Abstract

**Introduction:** The potential therapeutic efficacy of real-time fMRI Neurofeedback has received increasing attention in a variety of psychological and neurological disorders and as a tool to probe cognition. Despite its growing popularity, the success rate varies significantly, and the underlying neural mechanisms are still a matter of debate. The question whether an individually tailored framework positively influences neurofeedback success remains largely unexplored.

**Methods:** To address this question, participants were trained to modulate the activity of a target brain region, the visual motion area hMT+/V5, based on the performance of three imagery tasks with increasing complexity: imagery of a static dot, imagery of a moving dot with two and with four opposite directions. Participants received auditory feedback in the form of vocalizations with either negative, neutral or positive valence. The modulation thresholds were defined for each participant according to the maximum BOLD signal change of their target region during the localizer run.

**Results:** We found that 4 out of 10 participants were able to modulate brain activity in this region-of-interest during neurofeedback training. This rate of success (40%) is consistent with the neurofeedback literature. Whole-brain analysis revealed the recruitment of specific cortical regions involved in cognitive control, reward monitoring, and feedback processing during neurofeedback training. Individually tailored feedback thresholds did not correlate with the success level. We found region-dependent neuromodulation profiles associated with task complexity and feedback valence.

**Discussion:** Findings support the strategic role of task complexity and feedback valence on the modulation of the network nodes involved in monitoring and feedback control, key variables in neurofeedback frameworks optimization. Considering the elaborate design, the small sample size here tested (*N* = 10) impairs external validity in comparison to our previous studies. Future work will address this limitation. Ultimately, our results contribute to the discussion of individually tailored solutions, and justify further investigation concerning volitional control over brain activity.

## Introduction

The combination of mental imagery with neuromodulation techniques has received increasing interest in the field of translational research in clinical neuroscience (Skottnik and Linden, 2019). Neurofeedback is a neuromodulation technique that entails the self-modulation of specific brain regions or networks, through the “real-time” presentation of a representation of the ongoing brain activity, i.e., the participants are given information to enable mental imagery adaptive strategies (Megumi et al., 2015; Sitaram et al., 2016; De Vico Fallani and Bassett, 2019; Pamplona et al., 2020).

Despite the recent and extensive use of real-time functional Magnetic Resonance Imaging (rt-fMRI) neurofeedback, the underlying neural mechanisms subserving its cognitive components and its clinical impact is still the subject of an ongoing debate (Kadosh and Staunton, 2019; Paret et al., 2019). Learning to control brain activity is often associated with the identification of individually tailored mental strategies (Paret et al., 2018). The theory of reinforcement learning and operant conditioning has been discussed as model for neurofeedback mechanisms (Paret et al., 2018; Shibata et al., 2019), as the repetitive pairing of the target neural pattern and positive reward potentiates regional plasticity (Richards et al., 2019).

In a recent meta-analysis, Emmert et al. (2016) studied the various neural networks involved in neurofeedback. The authors describe a complex structure, most likely reflecting different cognitive processes, including reward processing and decision making (Haber and Knutson, 2010). These processes comprise the engagement of different networks, such as the central executive network, activated in cognitively demanding tasks, preparation and execution of mental strategies, and the saliency network, associated with attentional control and monitoring (Sridharan et al., 2008; Eckert et al., 2009).

Studies discriminating specific neural signatures associated with neurofeedback training have highlighted several cortical and subcortical structures, particularly key striatal subregions. Stronger ventral striatum activation has been associated to training success (Johnston et al., 2010). Recent evidence suggests that a network of cognitive, non-task specific, control regions, as well as regions implicated in reward and feedback monitoring were consistently activated during neurofeedback training (Skottnik et al., 2019). Volitional modulation of specific brain regions was accompanied by increased activation within the striatum, irrespective of the task. Altogether, these findings suggest that the reward network, particularly the striatum, plays a central role in neurofeedback training.

Most rt-fMRI neurofeedback paradigms aim for the decreasing or increasing of the activity within a specific brain region or functional connectivity between regions (Pereira et al., 2019; Pamplona et al., 2020). However, the heterogeneity of the results, the inter-subject variability of the efficacy and specificity of neurofeedback remains an important caveat (Linhartová et al., 2019; Paret et al., 2019). Strehl (2014) discussed the potential contribution of additional cognitive processes and related networks to boost the learning process associated with neurofeedback. The author highlights the impact of learning by trial and error and involved networks, cueing of behavior, feedback, reinforcement, and knowledge of results as well as transfer of volitional regulation skills. The acquisition of the self-modulation skill in neurofeedback studies has recently been addressed by Kadosh and Staunton (2019). Different elements, such as choice of feedback type, habituation and attentional variables, motivational aspects, and task engagement, appear to be implicated in the success of neurofeedback experiments, reported to be between 30 and 50% (Sokunbi et al., 2014; Kadosh and Staunton, 2019). The relevance of pre-training and experimental data in the definition of success has recently been discussed in (Haugg et al., 2020). Individual tailoring of the self-modulation framework may be key to the success of neurofeedback training.

The inter-subject variability of neurofeedback success and reported inability of some participants to achieve self-modulation even after multiple sessions undermines the efficiency of neurofeedback training and limits the translation to clinical populations. While neurofeedback has shown some promise, there are several caveats that need to be addressed to minimize the number of “non-responders,” optimize intervention efficacy and achieve its potential as a neurorehabilitation technique.

The motivation for this study was (i) to test the success rate of a novel interface based on explicit positive and negative reward and (ii) to investigate the impact of such an interface on the reward system. In this sense, the present contribution explores the neural correlates of feedback in rt-fMRI neurofeedback, regarding the reward and saliency networks, and the putative association with individual variations supporting the success of neurofeedback training. Moreover, it aims to characterize the influence of feedback valence, i.e., positive, neutral or negative auditory cues. An important feature for neurofeedback learning is to establish similarities and differences between neural patterns that process positive and negative reinforcement signals. Learning requires processing and interpretation of feedback as soon as it arrives (also described as feedback activity monitoring), and feedback evaluation considering the context of the desired outcome, i.e., estimation of a reward prediction error (Paret et al., 2018). In this sense, the evaluation of the distributed network involved in feedback interpretation is key. Accordingly, the notion of a distributed reward encoding network, involved in dopamine-based reinforcement learning, has been recently presented in Dabney et al. (2019).

This study, the third using the same core paradigm (Banca et al., 2015; Sousa et al., 2016), takes advantage of previous work on the self-modulation of the middle temporal visual area (hMT+/V5) complex activity level, a higher-order visual area shown to be responsive to both perceived and imagined visual information-based stimuli (Kaas et al., 2010). In our previous work, Banca et al. (2015) found evidence that hMT+/V5 can be volitionally modulated by focused imagery and the involvement of a specific cortico-cerebellar circuit. We have recently expanded these findings and demonstrated the feasibility of achieving more than two modulation levels in a single brain region (Sousa et al., 2016), by suggesting the use of three visual imagery tasks with different complexity. In Sousa et al. (2016), participants performed a functional localizer task designed to identify the region-of-interest (ROI) hMT+/V5 over the left and right regions involved in motion processing. This ROI served as the subsequent signal source for NF runs. Then, each subject performed four imagery runs with three different imagery tasks: stationary dot, dot moving in two directions, and dot moving in four directions. Real-time auditory feedback was computed at each time point based on the percentage of ROI mean signal change (PSC) in relation to the last baseline (down-regulation task) period. The feedback value was presented to the participant using auditory instructions to minimize the possibility to influence signals in hMT+/V5. The experimenter quantitatively forwarded changes in PSC, at every 4 s, from level 0—no activation to level 5—maximum activation. The results of the parametric activation paradigm, based on the increasing complexity of the imagery strategies, appear to further facilitate volitional control of target region brain activity when compared with standard up-regulation/down-regulation paradigms. These findings suggest that the complexity of the perceived challenge of the task positively influences the performance in neurofeedback, which is in accordance with previous studies (Kadosh and Staunton, 2019).

In this complementary, follow-up on our previous work on volitional neuromodulation of the hMT+/V5, we aimed to further investigate the reward system mechanisms involved in neurofeedback and the effect of feedback valence and task complexity on the neural correlates of neurofeedback training. We adopted an auditory feedback interface based on individually selected vocalizations and adaptive maximum modulation thresholds. We hypothesize that neurofeedback training with explicit reward cues leads to the differential activation profiles of cognitive control and feedback monitoring areas, i.e., we expect stronger task difficulty- and feedback valence-related neuroactivation in brain regions involved in neurofeedback training.

## Materials and Methods

### Sample

Ten healthy volunteers participated in this study (5 male), with ages between 19 and 35 years old (mean age = 26, SD = 5.25). One participant was left-handed. The inclusion criteria allowed participants with more than 18 years old without history of neurological or psychiatric disorders. Subjects were excluded in the case of any MRI contraindication. The study was approved by the Ethics Commission of the Faculty of Medicine of the University of Coimbra and was conducted in accordance with the Declaration of Helsinki.

### Study Design

**Figure 1:**
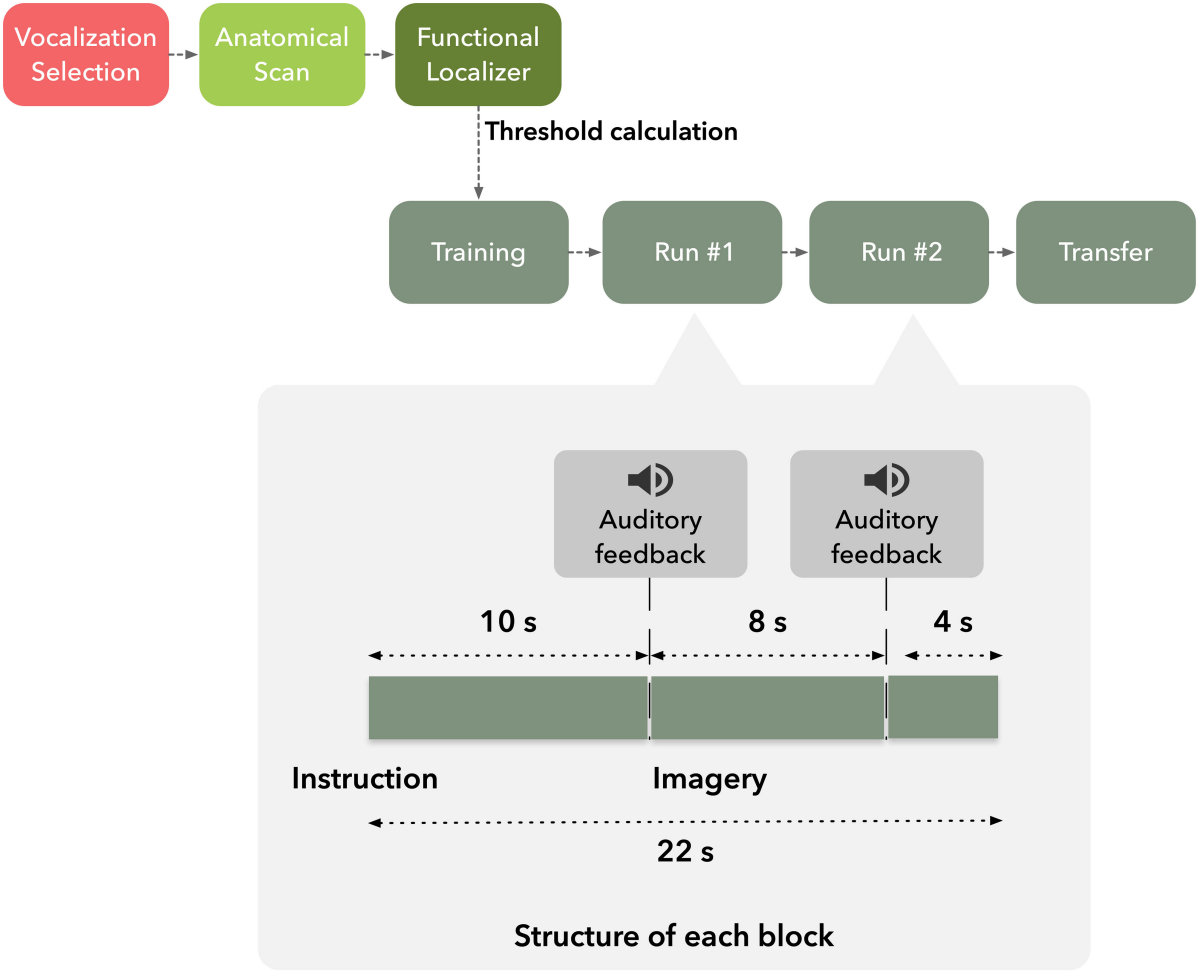
Graphical representation of the timeline of the experiment.

Each subject participated in a single neurofeedback session and was asked to volitionally modulate activity of the target brain region based on suggested visual imagery strategies (Figure 1). Moreover, participants were instructed that they would receive auditory feedback, based on pre-selected vocalizations, to assist them in two of four imagery runs. The participants were informed that the received vocalization would match the degree of success of self-modulation, e.g., positive vocalization if brain activation increased in up-regulation blocks. Furthermore, they were informed of the temporal delay of the Blood-Oxygen-Level-Dependent (BOLD) response and asked to minimize head movement during functional runs.

Each session started with the selection of 3 vocalizations: the most rewarding, most punishing, and a neutral, later used during neurofeedback training as auditory feedback. The identification of the vocalizations consisted of a two-stage questionnaire on a subset of 20 vocalizations from the database presented in Cowen et al. (2018). First, the subjects had to rate each vocalization from 1, most punishing, to 5, most rewarding. In a second questionnaire, participants were asked to only select one, in case of multiple vocalizations with maximum, minimum and median scores.

Subjects underwent an imaging session composed by T1-weighted anatomical scan followed by five functional runs in the order: hMT+/V5 localizer, neurofeedback training run, two neurofeedback and a transfer run.

Stimuli were presented on an LCD screen (70 x 39.5 cm^2^, resolution of 1,920 x 1,080 pixels, 60 Hz refresh rate) that the participants viewed through a mirror mounted above their eyes at an effective distance of 156 cm. The visual stimuli were based on a dot size of 0.5 x 0.5 cm^2^ (visual angle of the dot was 0.64 deg).

Scanning was performed using a 3T MRI scanner (Magnetom Prisma, Siemens, Erlangen, Germany) equipped with a 20-channel head coil, at the Portuguese Brain Imaging Network, University of Coimbra, Portugal. Functional images of the BOLD-contrast were acquired with a gradient echo T2^*^-weighted echo-planar-imaging (EPI) sequence. A sequence with repetition time (TR) 2 s was used (TE 30 ms, field-of-view 256 x 256 mm^2^, flip angle 90) with 33 slices and 4 x 4 x 3 mm^3^ voxel resolution. Anatomy was imaged with a 3D T1-weighted scan [Magnetization Prepared Rapid Acquisition Gradient Echo (MPRAGE) sequence, TE 3.42 ms, TR 2,530 ms, 176 slices, and field-of-view 256 x 256 mm^2^].

### Localizer Run

The functional localizer task was designed to delineate hMT+/V5, the neurofeedback target ROI (Banca et al., 2015; Sousa et al., 2016), and to define an individually-tailored threshold for feedback presentation.

The localizer run consisted of three active regulation conditions: two visual motion perception conditions, and one visual imagery condition. The visual motion perception conditions consisted of a moving white dot against a black background, either oscillating along a vertical trajectory (two opposite moving stimulus−2OMS) or vertical trajectory combined with a horizontal trajectory (four opposite moving stimulus−4OMS). The visual imagery condition consisted on the imagination of a dot moving in a vertical trajectory combined with a horizontal trajectory (four opposite motion imagery−4OMI). Each active regulation condition was randomly repeated four times, alternating with a stationary dot perception stimulus used as baseline—SS (13 repetitions). Participants were asked to look at a fixation cross in the center of the screen during the run. The run had a total of 25 blocks of 16 s, i.e., the overall duration of the functional localizer was 6 min and 40 s.

#### Neurofeedback Target Delineation

The definition of the neurofeedback target was performed in Turbo BrainVoyager 3.2 (TBV) (Brain Innovation, Maastricht, The Netherlands). The contrast of interest featured the balanced test 2OMS + 4OMS > baseline. A three-dimensional box was manually selected over the cluster displaying the strongest response in the statistical activation maps (defined according to *t*-statistic > 5) around bilateral middle temporal visual area.

#### Definition of the Feedback Presentation Threshold

Individual feedback-selecting thresholds (for the selection of auditory cue) were defined for each subject. Haugg et al. (2020) identified a positive correlation between pretraining activity levels and neurofeedback learning success. Our hypothesis is that task complexity and the difficulty level may represent an important factor in achieving self-modulation. In this sense, we aimed to determine individually tailored thresholds according to own self-regulation capabilities (represented in the functional localizer by the imagery condition). Our rationale was to define a threshold τ that would facilitate achieving positive auditory feedback during the most complex task [we hypothesize that activation in the target region would be higher, as previously established in Sousa et al. (2016)]. We calculated PSC for target region activity time course during the functional localizer and applied a 3-point time-window to smooth the time course and minimize the impact of outliers. The threshold τ was estimated as 50% of the maximum PSC value obtained during the 4OMI.

### Imagery Runs and Feedback Presentation

After the definition of the functional neurofeedback target mask, the participants performed four imagery runs, each composed by 275 volumes (lasting 9 min and 10 s each), divided in 25 blocks of 22 s duration. The first and last imagery runs (control runs) were performed without feedback.

Each run consisted of three conditions, two directions motion imagery−2OMI—and four directions motion imagery−4OMI, and a baseline, down-regulation condition, static imagery—SI. Six repetitions of the two active conditions (2OMI and 4OMI) were presented randomly (a total of 12 blocks). Each one of these blocks was flanked by SI blocks (13 blocks). We have previously established the static imagery task as a control, baseline task; in Sousa et al. (2016) SI would correspond to the baseline level in a parametric, 3-level, modulation effort. At the beginning of each block, auditory instructions informed the participants of the condition. Auditory feedback was presented in the second and third imagery runs through an interface based on three auditory cues consisting of vocalizations expressing positive, neutral and negative feedback. The auditory cue was presented at two specific timepoints within each block, 10 s and 18 s after block onset, as a function of the PSC time course. During the active, up-regulation, blocks, if the PSC was above the threshold τ the positive vocalization was played; the negative threshold was set to 0 (the rationale was to inform the participants that the modulation was in the opposite direction); if PSC was between 0 and the τ, the neutral cue was played. During the baseline, down-regulation, condition the goal was to lower the BOLD signal. To inform the participants of deviations (instability of the signal, either increase or decrease), we set the positive and negative thresholds to τ and −τ, respectively.

### Neuroimaging Data Analysis

#### Data Processing

Online data processing was based on Turbo-BrainVoyager v3.2 (Brain Innovation, Maastricht, The Netherlands). First, the functional mask for hMT+/V5 delineated based on the functional localizer run was loaded. Data processing included 3D motion correction, i.e., all functional volumes are aligned in space by rigid body transformation to the first recorded volume of the session. Data from the ROI were extracted in real-time and analyzed using custom MATLAB scripts to compute the corresponding auditory cue. Feedback was presented based on Psychtoolbox-3 routines (Brainard, 1997).

Offline processing was performed using BrainVoyager 21.2 (BV21.2) (Brain Innovation, Maastricht, Netherlands). Data processing included slice scan time correction, 3D motion correction and temporal filtering including linear trend removal and temporal high pass filtering with General Linear Model (GLM), and 2 Fourier Cycles. Functional data were co-registered with structural data of each participant and normalized to Talairach space. Finally, functional data were smoothed using an 8 mm kernel (full width at half maximum, FWHM) to account for between-subject variation in anatomical localization.

#### Statistical Analysis

In the first-level analysis of the functional runs, we defined a GLM for each run. The design matrix included a constant term and six realignment parameters as well as activity spike-related predictors. These parameters were obtained during motion correction and used to correct for movement-related artifacts not eliminated during realignment.

To assess the success in each run, we analyzed the statistical significance of the differences between up-regulation conditions and baseline within the neurofeedback target region (defined individually in bilateral occipito-temporo-parietal, hMT+/V5). To this end, we computed the ROI-GLM (the ROI for this analysis is the individual neurofeedback target) considering the contrast 2OMS + 4OMS > baseline. The statistical significance threshold was set to *p* = 0.05.

Second-level analysis of the functional data was performed to better understand the mechanisms involved in neurofeedback at the group level and to explore whole-brain patterns associated with the neurofeedback training. We performed a single factor, within-subject 3-level ANOVA analysis considering the conditions (i) SI, (ii) 2OMI, and (iii) 4OMI as levels. The *p*-values were adjusted based on the false discovery rate (FDR) to correct for multiple testing. The resulting statistical F-map was thresholded at q(FDR) = 0.05. The clusters extracted from this analysis had a minimum of 10 voxels.

In addition, we characterized temporal patterns of activity in the most relevant clusters. We focused particularly on clusters located in brain regions part of the feedback-related reward and saliency networks. The anterior insula and posterior cingulate cortex have been previously implicated in interoceptive and self-awareness processes associated with the salience network (Emmert et al., 2016). We will also consider the NF reward processing regions, such as the basal ganglia and anterior cingulate cortex (Paret et al., 2019). To this end, we computed the block-averaged response time-courses for each condition during the neurofeedback runs. Activation time courses were extracted, then averaged over the voxels in each ROI. For each run of each participant, the time-series were converted into PSC from average activity by dividing the signal measured at each time point by the average signal during the baseline, SI condition (i.e., positive values represent relative increases from the mean signal intensity in the stationary condition). The block-related responses for each condition were averaged across all participants from 2 s before to 24 s after each block onset (to fully cover the 22 s of each condition/block). We also characterized the temporal activation patterns associated with feedback events, in this case event-related responses for each feedback type (positive, neutral and negative) were averaged across all averages from 4 s before to 16 s after the auditory cue.

Finally, we determined the relation between the adaptive feedback threshold computed for each participant and the success in the neurofeedback run. To this end, we calculated the correlation coefficient.

## Results

### Functional Localizer

**Figure 2:**
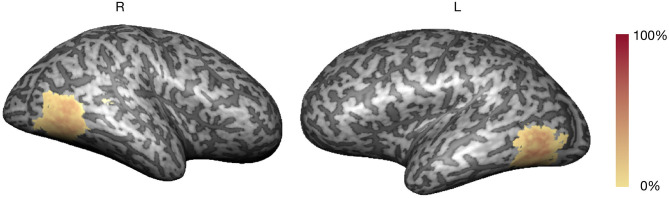
Neurofeedback target probability map (color scale: % overlap).

The localizer run allowed the real-time definition of subject-specific bilateral occipito-temporo-parietal ROIs selective for hMT+/V5, considering the balanced contrast (4OMS + 2OMS > SS). Figure 2 presents the probabilistic map of the ROI selection per participant, i.e., the color scale describes the probability of each voxel being selected as a neurofeedback target region.

The average volume of the neurofeedback mask was 3,030 ± 416 mm^3^ (maximum of 4,040 and minimum of 2,474 mm^3^). The clusters corresponding to the neurofeedback target were delineated in bilateral occipito-temporo-parietal, in accordance with previous studies (Sousa et al., 2016).

Pre-training data was used to characterize the subject's ability to modulate activity in the neurofeedback target region based on a visual imagery task. Mean threshold τ across subjects was 0.52 ± 0.16.

### Neurofeedback Runs

#### Neural Correlates of the Neurofeedback Training

**Figure 3:**
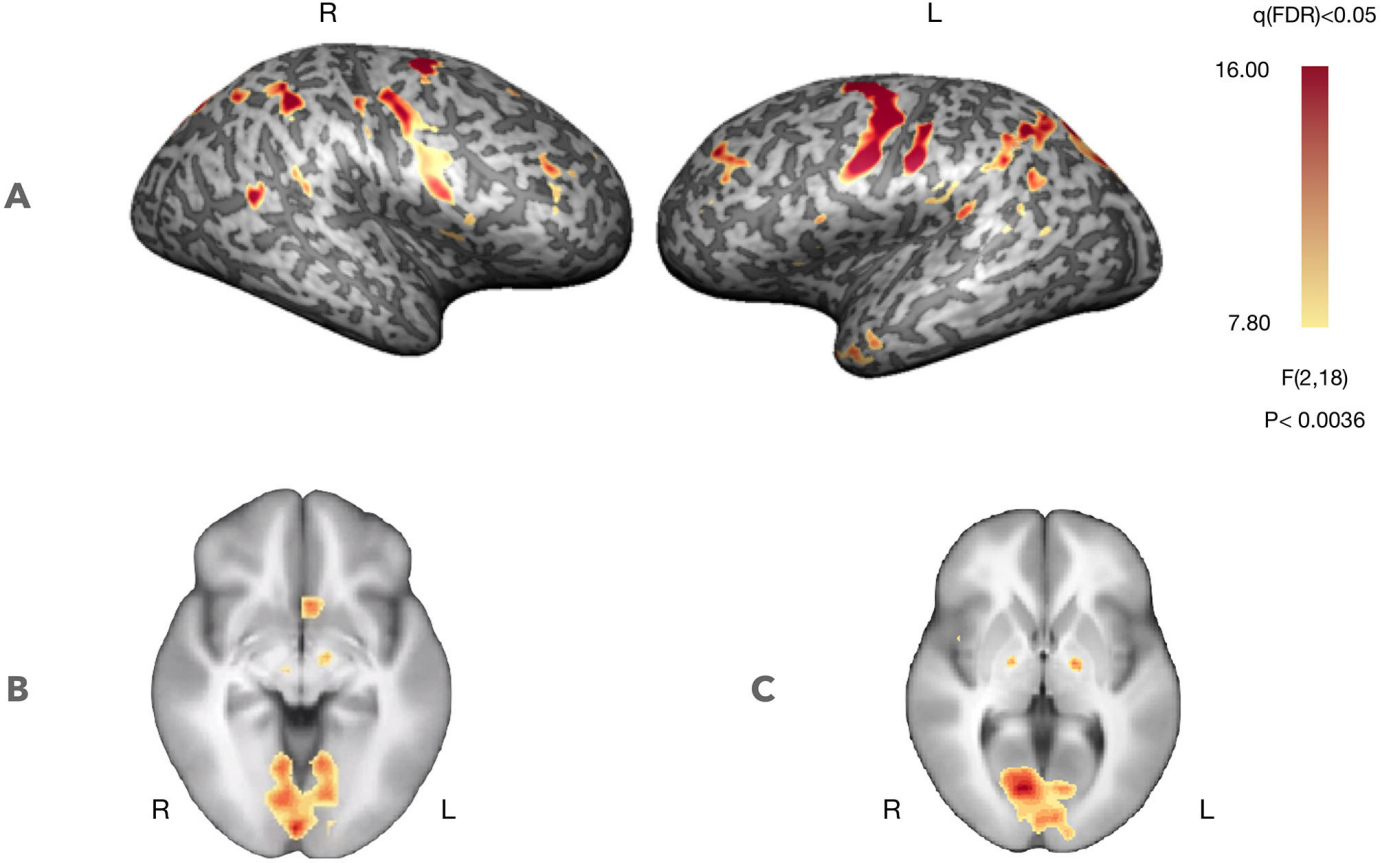
Self-regulation with neurofeedback (*N* = 10, considering both NF runs). Statistical maps for the ANOVA, 3-level within-subject analysis (FDR corrected q < 0.05) increased activation in prefrontal control regions and regions involved in feedback processing. **(A)** Statistical map projected on a participant's inflated cortex. **(B)** Statistical map projected on an average of the individual anatomical data sets (radiological convention)—ventral striatum (*Z* = −5, Talairach coordinates). **(C)** Statistical map projected on an average of the individual anatomical data sets (radiological convention)—dorsal striatum (*Z* = 3, Talairach coordinates).

Figure 3 presents whole-brain group activation maps (*N* = 10) for the neurofeedback runs. Table 1 summarizes the clusters of voxels that were found with the ANOVA 3-level within-subject factor analysis (representing the three conditions). The clusters represent the brain regions involved in the participants' effort to modulate brain activity in the target region. The threshold was set at *q* = 0.05, FDR corrected. Clusters associated with the proposed neurofeedback task were found bilaterally in the middle frontal gyrus, lentiform nucleus, superior temporal gyrus, right precuneus, right posterior cingulate cortex, left precentral gyrus, and left caudate and nucleus accumbens.

**Table 1:** Brain areas with significant activation, considering the 3-factor ANOVA analysis (FDR corrected q < 0.05), during neurofeedback runs.

**Talairach coordinates**			**Size (mm^**3**^)**	**Hemisphere**	**Anatomy (Brodmann area, BA)**	**z-score**
**X**	**Y**	**Z**				
60	−40	19	1,172	Right	Superior temporal gyrus (22)	11.717645
24	−13	49	9,436	Right	Precentral gyrus (6)/inferior frontal gyrus (44)/anterior insula (13)	12.356137
18	−67	46	13,485	Right	Precuneus (7)	14.198732
45	35	34	739	Right	Dorsolateral prefrontal/middle frontal gyrus (9)	9.332857
15	−7	4	325	Right	Thalamus/striatum (putamen)	8.646475
3	−52	28	1,239	Right	Posterior cingulate gyrus (23, 31)	9.179523
−24	−13	52	10,913	Left	Precentral Gyrus (6)	19.054468
−3	14	−2	581	Left	Striatum (caudate, nucleus accumbens)	9.211643
−18	−82	40	19,627	Left	Precuneus (7), cuneus (19)	11.838016
−21	−4	13	1,553	Left	Striatum (putamen)	9.363536
−15	−25	13	629	Left	Thalamus	9.646857
−36	44	37	1,820	Left	Dorsolateral prefrontal/middle frontal gyrus (9)	10.390186
−41	−1	−20	1,165	Left	Temporopolar area/superior temporal gyrus (38)	9.877755
−48	5	19	901	Left	Inferior frontal gyrus (44)/anterior insula (13)	9.468178
−48	−16	−17	1,527	Left	Middle temporal gyrus (21)	10.016431

*Provided coordinates are in Talairach space*.

#### Neurofeedback Target Modulation

We assessed whether subjects were able to regulate the activity of hMT+/V5 during the neurofeedback runs. A repeated measure ANOVA determined that neurofeedback target ROI mean activity did not differ significantly between up-regulation conditions (2 or 4 fold motion) conditions [*F*_(2, 18)_ = 0.527, *p*-value = 0.599].

**Figure 4:**
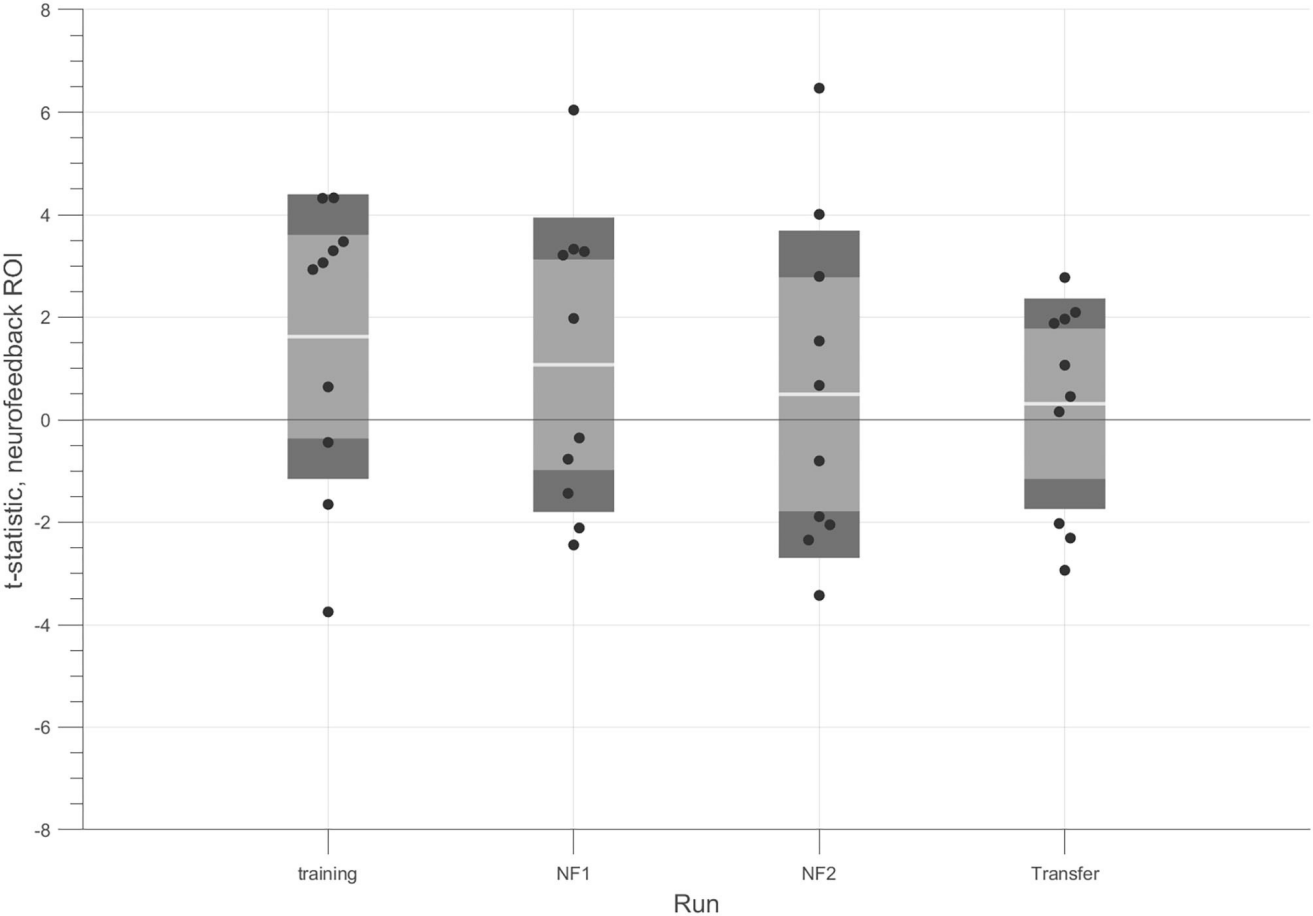
*t*-tests for each run considering the contrast (4OMI + 2OMI) > SI within hMT+/V5 ROI. Black dots correspond to the *t*-statistic of each participant for the contrast of interest. The white line corresponds to the mean value across participants, the light gray area represents the standard error of the mean (mean ± SEM), and the dark gray regions correspond to the standard deviation (mean ± SD).

We also analyzed the pattern across runs. Figure 4 shows the modulation results for each run. The *t*-tests for the contrast 4OMI + 2OMI > SI suggest a trend for a slight decrease of success (at group-level) throughout the experiment.

An individual analysis demonstrated that 4 participants successfully modulated the target ROI during the neurofeedback runs (we used a *t*-test 4OMI + 2OMI > SI to determine modulation success, *p*-value < 0.05).

**Figure 5:**
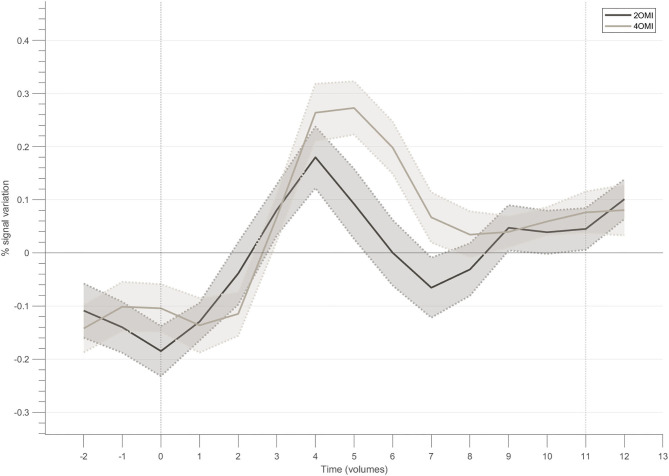
Block-related responses averaged across the participants that successfully modulated the ROI within the neurofeedback target.

Figure 5 presents the block-related responses to each condition averaged across the 4 participants that successfully modulated the ROI, considering the NF target. The selection of this subset of participants allows us to better understand the modulation pattern for each condition when successfully performing the task (with different difficulty levels). In this subset of 4 participants, the response pattern for the motion imagery conditions, i.e., 4OMI and 2OMI, presents an increase, reaches a peak and then a slight decrease to a plateau. The peak for the 4OMI condition is higher than the one present for the condition 2OMI, as found in Sousa et al. (2016).

#### Relation Between Threshold and Neurofeedback Success

**Figure 6:**
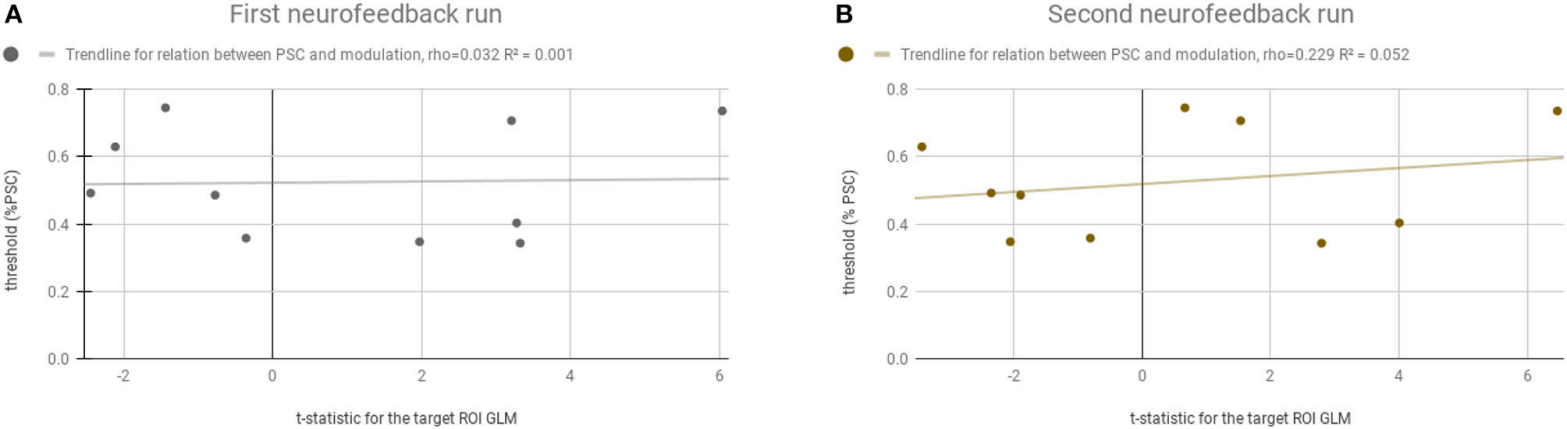
Relation between the computed adaptive threshold and the success in the neurofeedback runs. Y-axis represents the threshold (% PSC) selected for each participant and the x-axis represents the t-statistic for the ROI-GLM contrast (within the neurofeedback target) (4OMI + 2OMI < SI). **(A)** Represents the first neurofeedback run. **(B)** Represents the second neurofeedback run.

Figure 6 shows that there is no systematic relation, or bias, between the adaptive feedback threshold computed for each participant and the success in the neurofeedback runs. The threshold (y-axis) ranged from 0.34 to 0.74%. The *t*-statistic (x-axis) from the balanced contrast 2OMI + 4OMI > SI did not vary as a function of the threshold selected, i.e., the ability to modulate brain activity in the target region was not related with the selection of the threshold required to receive positive and negative feedback. The Pearson correlation between neurofeedback modulation ability (as measured by the contrast of interest) and the individual threshold selected was 0.032 and 0.229 (non-significant, n.s.), first and second neurofeedback run, respectively.

#### Block-Related Responses—The Neural Correlates of Feedback

**Figure 7:**
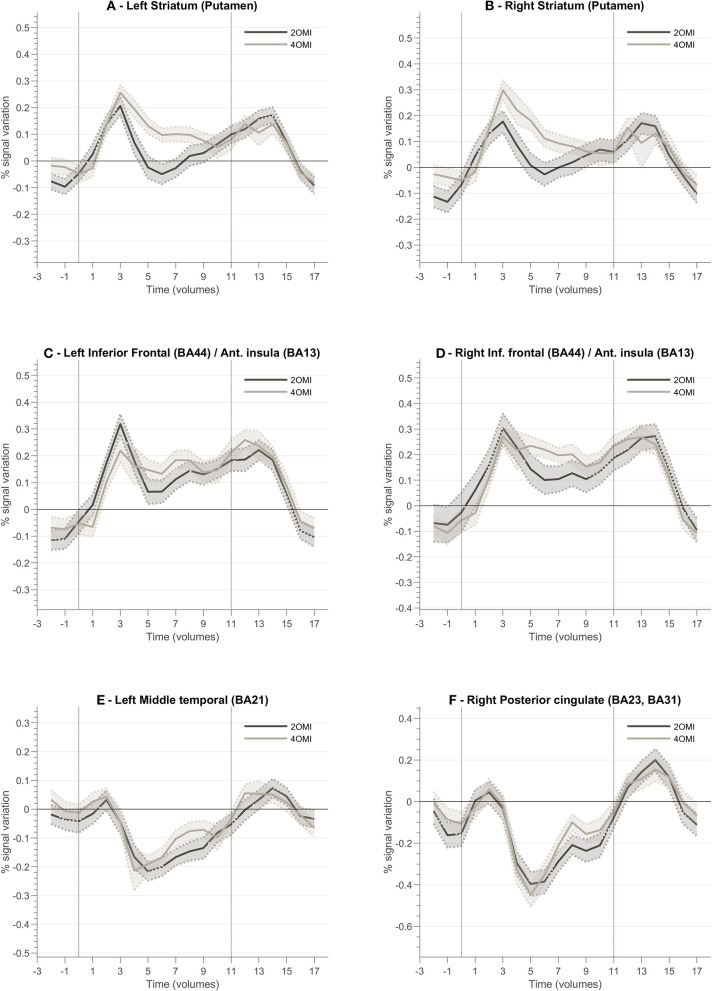
Block-related responses averaged across all participants for different ROI involved in the neurofeedback task and identified based on ANOVA 3-level within-subject factor analysis. Each plot presents the block-related curve for the conditions 2OMI and 4OMI for the following ROIs: **(A)** Left Striatum (Putamen); **(B)** Right Striatum (Putamen); **(C)** Left Inferior Frontal Cortex (Brodmann Area, BA, 44)/Anterior Insula (BA13); **(D)** Right Inferior Frontal Cortex (BA44)/Right Anterior Insula (BA13); **(E)** Left Middle Temporal Cortex (BA21); **(F)** Right Posterior Cingulate Cortex (BA23, BA31).

In order to characterize the regions involved in the processing of feedback, we studied their average BOLD responses throughout neurofeedback runs. To this end, we selected clusters located in brain regions part of the feedback-related reward saliency and valence networks. Figure 7 presents the block-related activation patterns of these structures across all participants. This analysis comprises all participants as we aim to explore how this novel feedback approach was interpreted irrespective of the success. We included both neurofeedback runs in this analysis. Baseline was computed based on the signal from the static imagery condition (down-regulation condition). Left and right striatum (Figures 7A,B) (putamen) show an increase of activity during the motion imagery conditions. Additionally, we note that the response is greater for the 4OMI condition compared to 2OMI. This pattern is also noticeable but less evident in areas connected to left and right anterior insula (Figures 7C,D). The pCG and the left middle temporal gyrus present a deactivation during the motion imagery conditions (Figures 7E,F).

#### Interpreting the Valence of the Feedback—Positive vs. Negative Events

In order to better understand the correspondence of feedback valence and the BOLD pattern in the network activated during the neurofeedback task, we calculated the event-related responses for positive and negative feedback. First, to compute PSC we considered the entire run average as baseline. Event-related responses associated with the feedback events, i.e., positive and negative vocalizations, were determined as the average across all participants from 4 s (2 volumes) before to 16 s (8 volumes) after each event, i.e., onset of the presentation of the vocalization.

**Figure 8:**
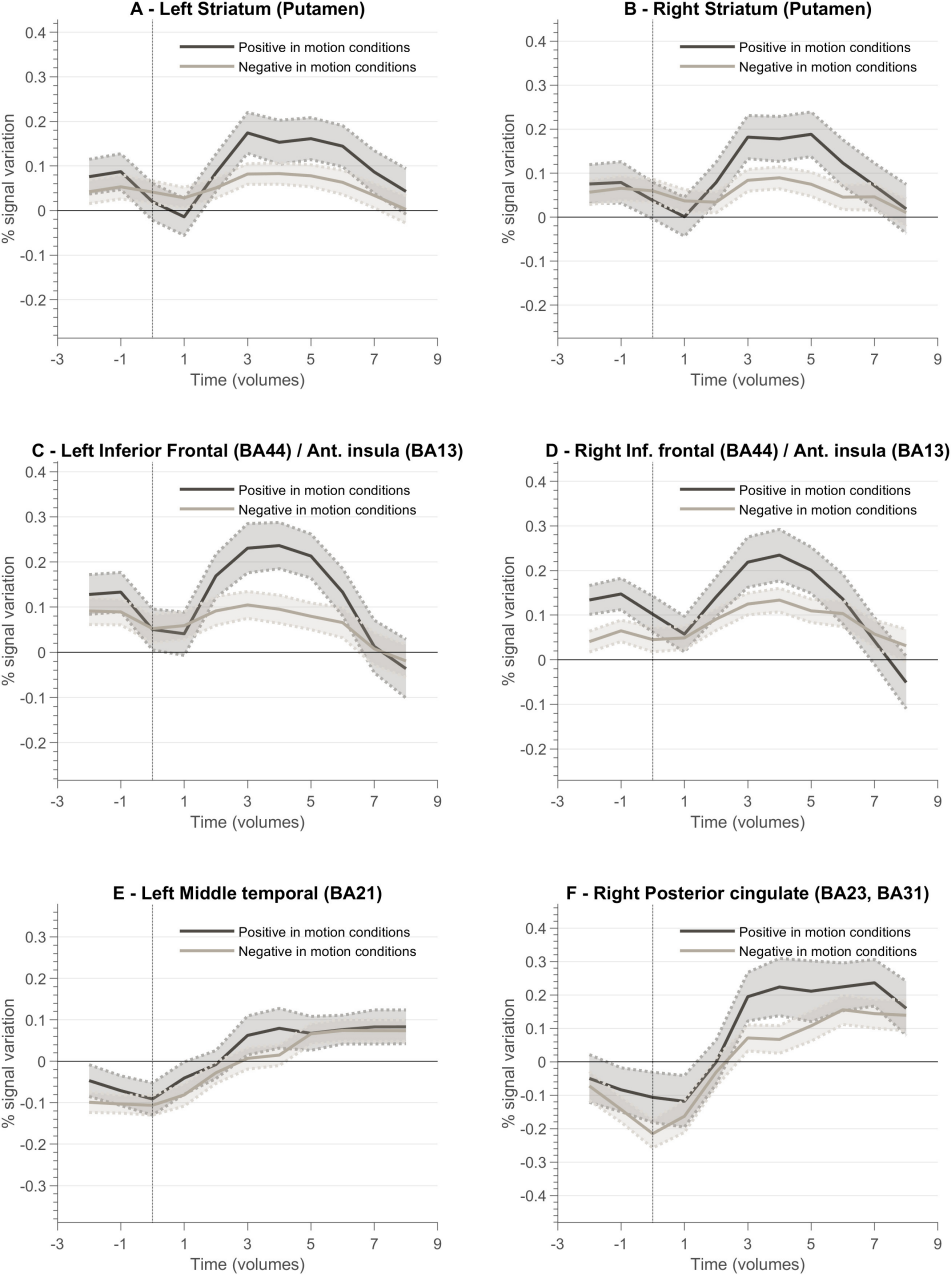
Feedback valence—(positive and negative) event-related responses averaged across all participants (n = 10) for different ROIs involved in the neurofeedback task. Each plot presents the positive and negative feedback event-related curve for the following ROIs: **(A)** Left Striatum (Putamen); **(B)** Right Striatum (Putamen); **(C)** Left Inferior Frontal Cortex (Brodmann Area, BA, 44)/Anterior Insula (BA13); **(D)** Right Inferior Frontal Cortex (BA44)/Right Anterior Insula (BA13); **(E)** Left Middle Temporal Cortex (BA21); **(F)** Right Posterior Cingulate Cortex (BA23, BA31).

**Figure 9:**
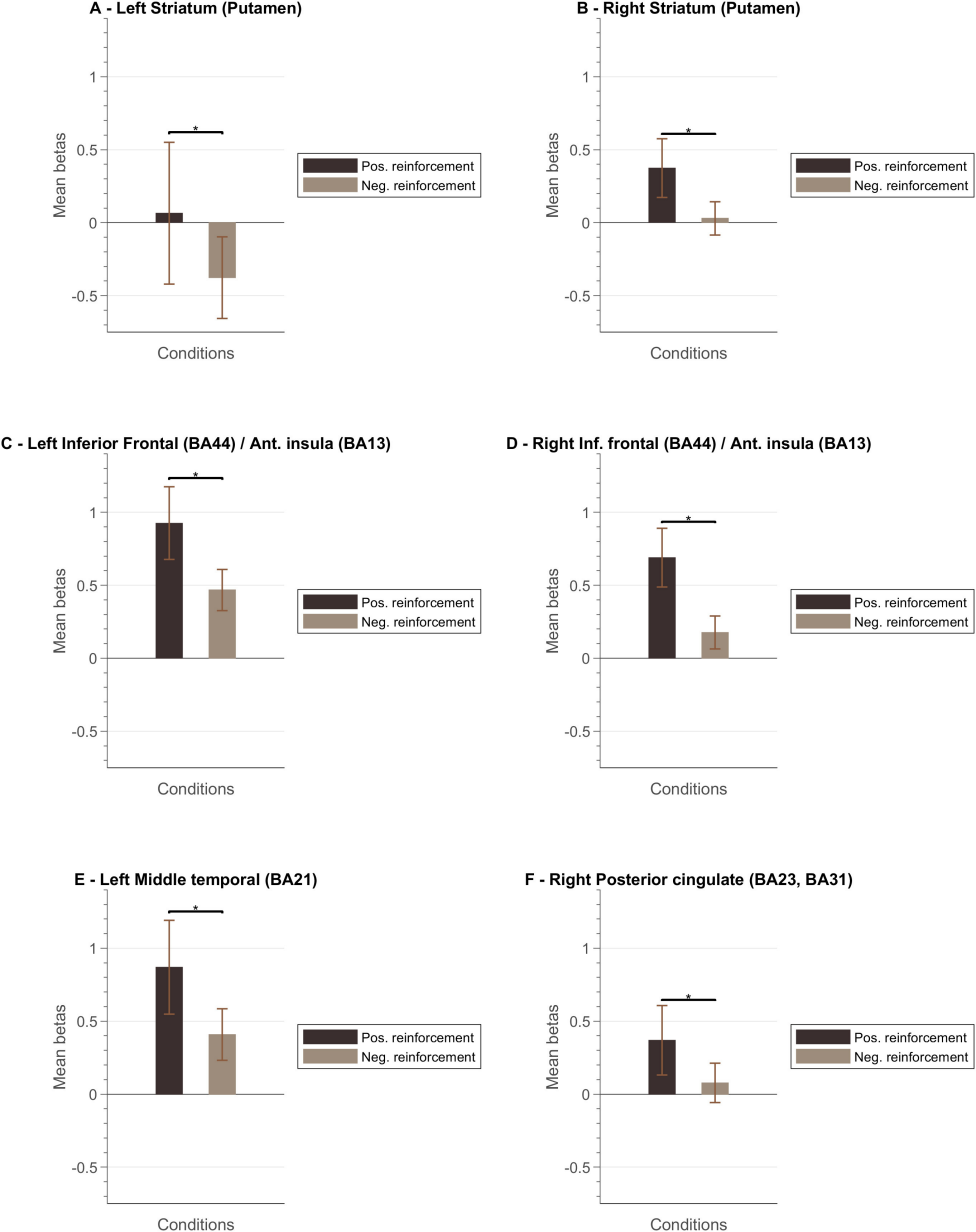
Mean beta values per ROI. The left columns for each ROI correspond to the mean bata value associated to the “positive reinforcement” events and the right columns to the “negative reinforcement” events (the whiskers represent the SEM) for the following ROIs: **(A)** Left Striatum (Putamen); **(B)** Right Striatum (Putamen); **(C)** Left Inferior Frontal Cortex (Brodmann Area, BA, 44)/Anterior Insula (BA13); **(D)** Right Inferior Frontal Cortex (BA44)/Right Anterior Insula (BA13); **(E)** Left Middle Temporal Cortex (BA21); **(F)** Right Posterior Cingulate Cortex (BA23, BA31).

Figure 8 presents a summary of the results. In general, the ventral and dorsal striatum, and anterior insula tend to present stronger activation patterns for positive feedback cues compared to negative feedback. To complement this data, we also designed GLM with feedback event-related regressors and analyzed the resulting betas per ROI of interest. Figure 9 summarizes mean beta values for each ROI (which are directly related to PSC, considering a percent-transform time course normalization).

## Discussion

In this experiment, we sought to investigate the neural correlates of feedback-related reward saliency and valence during fMRI-based neurofeedback training, when feedback explicitly carried individually tailored reward signals.

In this study, we propose the introduction of a new feedback interface that aims to optimize current approaches based on recent findings on the neural correlates of neurofeedback (Skottnik et al., 2019) and neurofeedback learning theories (Strehl, 2014; Wood et al., 2014). In this sense, the motivation for this study was (i) to test the success rate of a novel interface based on explicit positive and negative reward and (ii) to investigate the impact of such an interface on the reward system.

To this end, we based our paradigm on a previously validated experiment, self-modulation of the hMT+/V5 (Banca et al., 2015; Sousa et al., 2016), and adapted the feedback with individually selected vocalizations for feedback presentation (with negative, neutral, and positive interpretation). Additionally, we used information from the localizer run to select the participant specific threshold to determine the valence of the feedback. In this way, we aimed to isolate the neural network involved in neurofeedback-driven self-modulation and characterize the responses to positive and negative reinforcement.

The localizer run allowed us to successfully identify bilateral hMT+/V5, our target region, in all participants; the results were in accordance with the findings presented in Sousa et al. (2016).

As in our previous work, we found evidence that self-modulation of BOLD activity in hMT+/V5 can be achieved using the same strategy across participants. Our results (40% of participants were able to modulate activity during the neurofeedback runs) are inferior to the ones previously reported (75% in Banca et al., 2015; and 85% in Sousa et al., 2016). Although the adjustments made in the study design and feedback paradigm address our objective to investigate the reward system relation to neurofeedback, it showed a decrease in neurofeedback efficacy, i.e., less “learners” and some evidence for deterioration throughout the session. The comparison between the *t*-values associated with the contrast of interest between the current and previous study (Sousa et al., 2016) shows that, on average, participants performed worse here than in our previous study. These results suggest that explicit reward as presented (feedback schedule, auditory cues selected, etc.) in this design, may worsen the ability to control volitional modulation of the activity in the neurofeedback target region. A recent study found a beneficial effect of combining reinforcement and punishment, i.e., positive and negative feedback (Klöbl et al., 2020). The authors emphasize the possible role of error avoidance as a complementary mechanism to improve neurofeedback-based learning. Our results do not confirm this hypothesis and warrants further investigation concerning its role in neurofeedback success.

Additionally, a sparser presentation of feedback and interpretability of the auditory feedback cues may also contribute to these results. In contrast to the current study, the setup in Sousa et al. (2016) presented a simpler, numerical based auditory feedback each 2 TRs.

Debriefing from the participants of this study identified that vocalization-based feedback may be distracting, and that feedback schedule may have contributed to the limited success of the interface. Altogether, our results (imaging and reporting from the participants' debriefing) may suggest an unsuccessful compromise between informative and positive/negative reinforcement cues. Wood et al. (2014) suggests a compromise between feedback information regarding automatic processes and information to engage cognitive activity operating under conscious control, ultimately a more selective schedule of reward and punishment.

The characterization of the block-related responses within the neurofeedback target region of the successful modulators shows that the visual motion imagery strategies used by the participants evoked differential brain responses according to the number of imagined motion variations, replicating previous findings Sousa et al. (2016). The average time course per condition shows that the participants were able to elicit different patterns for the three conditions, e.g., activation for the 4OMI > 2OMI as hypothesized. The suggested task (i.e., 2OMI vs. 4OMI) had a differential impact on the activation patterns of different brain regions while performing neurofeedback training. Our results also suggest that feedback valence had a different impact on specific brain nodes of the network commonly involved in neurofeedback success.

Additionally, we found that threshold selection based on the ability of each subject to imagine the moving dot during the localizer run was not associated with the decrease of success in neurofeedback runs.

### Neural Correlates of Neurofeedback

The ANOVA 3-level within-subject analysis highlighted a set of clusters associated with the performance of the neurofeedback task including bilateral middle frontal gyrus, lentiform nucleus, superior temporal gyrus, insula, precuneus, right posterior cingulate cortex, left precentral gyrus, and left nucleus accumbens. The presence of these structures is in accordance with our previous findings and recent studies on reinforcement and punishment in learning dynamics (Banca et al., 2015; Klöbl et al., 2020).

The simultaneous involvement and balance between several networks in neurofeedback tasks is one important aspect to consider in training success. Different structures highlighted here may have significant roles in this respect. The role of the anterior insula and the basal ganglia in neurofeedback has been previously reported in several studies [see meta-analysis presented in Emmert et al. (2016)]. Anterior insula activation, particularly within the right hemisphere, has been associated with coordination and evaluation of task performance (Eckert et al., 2009; Menon and Uddin, 2010). The authors also discuss the importance of the insula and basal ganglia in motivational processing, a key aspect of neurofeedback training. Ventral striatum is also part of the reward network, as reward-related activity in ventral striatum has been demonstrated in FitzGerald et al. (2014).

The anterior cingulate cortex role in the regulation network and involvement in error monitoring processes have also been previously discussed (Sokunbi et al., 2014; Paret et al., 2019). The activation of the middle Frontal gyrus (dorsolateral prefrontal cortex) is often associated with regulation processes involved in neurofeedback (Zotev et al., 2013; Japee et al., 2015). These results are also consistent with previous studies implicating these regions with error monitoring (Carter et al., 1998; Menon et al., 2001).

Additionally, our analysis showed a deactivation of several regions including the posterior cingulate cortex and middle temporal gyrus. These regions, part of the default mode network (DMN), have been previously reported to deactivate when performing cognitively demanding tasks (Emmert et al., 2016).

Together, this set of regions form a network of control/regulation and reward processing, is in line with the conceptualization presented, for example in Paret et al. (2019) and Skottnik et al. (2019).

### Role of Context, Reward Processing, and Feedback Valence

To better understand the role of different brain regions, we explored the block-related average per condition of the BOLD activity. Left and right striatum (putamen) ROIs present a different pattern between conditions (2OMI vs. 4OMI). The block-related response for the more complex (4 motion) imagery strategy elicits a higher BOLD activity variation. This pattern is also present in the right anterior insular cortex. The results suggest that these regions are sensitive to the context (task complexity) and/or expected reward. One reason for this difference could be the relative context, provided by the specific strategy, in part because of the effect of task difficulty in the representation and encoding of feedback (Holroyd et al., 2004; Padrón et al., 2016; Steel et al., 2019). The authors concluded that the functional changes associated with reward and punishment are associated with the context. In FitzGerald et al. (2014), the authors also found that ventral striatum activity during decision making is dynamically modulated by context.

We also analyzed the event-related response associated with the valence of the feedback, i.e., type of vocalization. Again, we aimed at evaluating the encoding of valence in the brain ROIs associated with neurofeedback self-modulation, particularly the regions that are affected by the context/task complexity. We found that reward and punishment (positive and negative feedback) differentially impacted BOLD response in bilateral putamen, insula, and left ventral striatum. These results are in line with previous studies suggesting that neural areas supporting learning present functional differences associated with how feedback information is presented (Bischoff-Grethe et al., 2009). Our results show that positive feedback drives a stronger BOLD response, while negative feedback present a weaker response in limbic areas. We also identified a stronger response to positive vocalization in insula ROIs, i.e., insula presented a stronger engagement when the reinforcement is positive. While the activity of the insula has been previously associated with negative stimuli such as pain (Xu et al., 2020), our results suggest that insula activity patterns in neurofeedback experiments may be driven by the emotional response to (positive) reward explicit feedback. This is particularly relevant in the context of this study because feedback and reward are combined. Moreover, these results are in accordance with reports highlighting anterior insula role in the modulation of neural hubs that underlie several processes (Lubianiker et al., 2019).

Recently, Klöbl et al. (2020) also studied insula activation during regulation periods but did not report significant differences between negative and positive reward events. Differences between studies may result from activation of different sub-regions of the insula. Altogether, these results complement the discussion on the motivation for novel interfaces and highlight the importance of reward-based feedback. However, the lower level of success (comparing the neuromodulation scores achieved in this study with previous approaches) suggests an unbalance between informative and positive/negative reinforcement cues. Future work could explore the combination of both types of feedback—e.g., informative during the conditions and reinforcement at the end of each trial, probing reward-related processes and cognitive control mechanisms. Ultimately, we hypothesize that the fine tuning of this combination may optimize neurofeedback success, although we recognize that in our case this was not successful at the group level.

### Limitations

In this study we add three different manipulations to the neurofeedback training: adaptive threshold (with the goal of tailoring NF for each individual), task complexity (to replicate previous findings and extend them to the context of the goals of the current study), and feedback interpretation based on positive, negative or neutral vocalizations. To disentangle the role of each node in the network involved in e.g., feedback monitoring, task performance, control/attention is not trivial and should be interpreted in the context of an exploratory study (Paret et al., 2018). Moreover, a specific control group receiving different feedback cues would be necessary to validate the benefits of feedback based on positive or negative reinforcement cues. In this sense, the relatively small sample size is a major limitation in the interpretation of the results. No sham group was included as the main goal was to explore the specific, individual mechanisms associated with feedback valence and task complexity. Nevertheless, as this study represents a follow-up on our previous work, the analyses were based on previous knowledge on the mental strategies, neurofeedback target region (Banca et al., 2015; Sousa et al., 2016), and on a subject-specific approach to explore the temporal activation pattern. Even though the results only partially overlap with our previous findings, as we clearly see a decrease in neurofeedback training success, the activation patterns in different reward- and control- network structures are in line with previous studies.

## Conclusion

Our study contributes to the understanding of current neural models of neurofeedback. The results here presented suggest that task complexity/context and feedback valence have an important role in the modulation of the networks nodes involved in monitoring and control of feedback, highlighting their importance in learning of voluntary neuromodulation. Although the current paradigm was particularly valuable in assessing positive and negative reward related responses, this was achieved at the cost of relatively lower efficacy of NF than the previous study of Sousa et al. (2016). On the one hand, our results suggest that explicit reinforcement feedback may play a crucial role (different patterns were found in several brain structures involved in neurofeedback training). On the other hand, results of self-modulation ability of the target region and anecdotal reporting from the participants' debriefing may indicate an unsuccessful compromise between informative and positive/negative reinforcement cues. Future analyses should explore the role of these areas—e.g., study the relation between top-down and bottom-up mechanisms during the feedback events and neurofeedback success, establishing the functional networks involved in feedback valence and task complexity. In this sense, recent studies have explored the potential impact of gaining control of specific components of brain networks (Bassett and Khambhati, 2017; De Vico Fallani and Bassett, 2019; Pamplona et al., 2020).

Ultimately, this information may allow the development of individually tailored frameworks for neurofeedback, providing the ground for definition of potential neural framework for neurorehabilitation success.

## Data Availability Statement

The raw data supporting the conclusions of this article will be made available by the authors, without undue reservation.

## Ethics Statement

The studies involving human participants were reviewed and approved by Local ethical committee of Faculty of Medicine, University of Coimbra, Portugal. The patients/participants provided their written informed consent to participate in this study.

## Author Contributions

BD, MR, AS, JP, TS, and MC-B discussed and conceived the study and discussed the results. BD, MR, and MC-B designed the study. MR performed the recruitment. MR, BD, JP, and AS acquired and analyzed the data. BD and MR wrote the manuscript. JP, AS, TS, and MC-B reviewed the manuscript. All authors read and approved the final manuscript.

## Conflict of Interest

AS was a recipient of a PhD scholarship by Siemens Healthineers, Lisbon, Portugal. The remaining authors declare that the research was conducted in the absence of any commercial or financial relationships that could be construed as a potential conflict of interest.
